# Imaging the local biochemical content of native and injured intervertebral disc using Fourier transform infrared microscopy

**DOI:** 10.1002/jsp2.1121

**Published:** 2020-09-14

**Authors:** Stephen R. Sloan, Christoph Wipplinger, Sertaç Kirnaz, Robert Delgado, Steven Huang, Gennady Shvets, Roger Härtl, Lawrence J. Bonassar

**Affiliations:** ^1^ Meinig School of Biomedical Engineering Cornell University Ithaca New York USA; ^2^ Department of Neurological Surgery Weill Cornell Medical College New York New York USA; ^3^ Applied Engineering and Physics Cornell University Ithaca New York USA; ^4^ Sibley School of Mechanical and Aerospace Engineering Cornell University Ithaca New York USA

**Keywords:** collagen, degeneration, Fourier transform infrared microscopy, intervertebral disc, proteoglycan

## Abstract

Alterations to the biochemical composition of the intervertebral disc (IVD) are hallmarks of aging and degeneration. Methods to assess biochemical content, such as histology, immunohistochemistry, and spectrophotometric assays, are limited in their ability to quantitatively analyze the spatial distribution of biochemical components. Fourier transform infrared (FTIR) microscopy is a biochemical analysis method that can yield both quantitative and high‐resolution data about the spatial distribution of biochemical components. This technique has been largely unexplored for use with the IVD, and existing methods use complex analytical techniques that make results difficult to interpret. The objective of the present study is to describe an FTIR microscopy method that has been optimized for imaging the collagen and proteoglycan content of the IVD. The method was performed on intact and discectomized IVDs from the sheep lumbar spine after 6 weeks in vivo in order to validate FTIR microscopy in healthy and degenerated IVDs. FTIR microscopy quantified collagen and proteoglycan content across the entire IVD and showed local changes in biochemical content after discectomy that were not observed with traditional histological methods. Changes in collagen and proteoglycans content were found to have strong correlations with Pfirrmann grades of degeneration. This study demonstrates how FTIR microscopy is a valuable research tool that can be used to quantitatively assess the local biochemical composition of IVDs in development, degeneration, and repair.

## Graphical abstract

In this study, we present a method for quantitatively assessing the spatial distribution of biochemical contents within the intervertebral disc using Fourier transform infrared (FTIR) microscopy. FTIR microscopy quantified collagen and proteoglycan content across the entire IVD and showed local changes in biochemical content after discectomy that were not observed with traditional histological methods. This study demonstrates how FTIR microscopy is a valuable research tool that can be used to quantitatively assess the local biochemical composition of IVDs in development, degeneration, and repair.

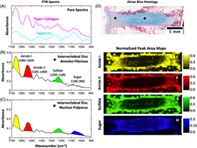

## 1 INTRODUCTION

Intervertebral discs (IVDs) are a composite tissue in the spine primarily consisting of proteoglycans in the nucleus pulposus (NP) and a fibrous collagen network in the annulus fibrosus (AF).^1^ The proteoglycan and type‐II collagen content of the IVD decrease radially from the NP to AF, while type‐I collagen content increases from the inner to outer AF.^1^, ^2^ Proteoglycans imbibe water and create hydrostatic pressure within the AF, which contains the pressure via hoop stresses generated in its fibrous, lamellar collagen network.^3^ Thus, proteoglycans within the NP and an intact, fibrous AF are critical to the mechanical function of the IVD. As IVDs age, encounter traumatic injuries or undergo degenerative changes, native disc cells become quiescent and lose their ability to maintain the intricate extracellular matrix (ECM).^4^, ^5^ Also, disc and immune cells begin produce matrix‐degrading enzymes that catabolize collagens and proteoglycans. Local changes to the biochemistry can ultimately lead to additional macroscale changes to the IVD such as NP dehydration, AF lesions and loss of disc height that reduce functionality, cause pain and are indications for surgical interventions such as discectomy or fusion.^6^, ^7^ As such, investigating the biochemical composition of IVDs in development, disease progression, and after therapeutic intervention is necessary to improve the quality of spine healthcare.

There is a need in the research community for an analytical method that can quantitatively measure the local distribution of biochemical components of the IVD without a priori knowledge of what to look for. Traditional biochemical analysis methods such as histology, immunohistochemistry (IHC) and spectrophotometric assays are valuable experimental tools, but the user must know exactly what they are looking for and no single method can quantitatively analyze the spatial distribution of biochemical components with high resolution. Fourier transform infrared (FTIR) microscopy and Raman microscopy are two vibrational spectroscopy methods that have received interest from many fields studying soft tissues for their ability to gather both quantitative and spatial data about biochemical composition.^8^, ^9^, ^10^, ^11^, ^12^, ^13^, ^14^, ^15^, ^16^, ^17^, ^18^, ^19^, ^20^ FTIR microscopy uses infrared (IR) light to probe the vibrational and rotational modes of both organic and inorganic molecules. When broad‐spectrum IR light is passed through a molecule, light is absorbed specifically at the frequency of the atomic vibrations and rotations. Since every known molecule has a unique atomic composition and bond structure, each molecule has a unique IR absorbance spectra that is characterized by absorbance peaks at specific vibrational and rotational frequencies. In addition, Beer's law states that IR absorbance is proportional to molecular concentration, and that a mixed species of molecules have additive contributions to the combined IR absorbance.^21^ In FTIR microscopy, FTIR spectra are collected from a defined area on unstained histologic sections.

FTIR microscopy has been used over the past two decades to study the composition of biological tissues like bone and cartilage.^8^, ^10^, ^14^, ^15^, ^16^, ^17^, ^18^, ^22^ The technique lends itself to studying these tissues because the primary constituents, collagen and proteoglycans, have FTIR absorbance spectra with distinct peaks.^23^ The articular cartilage community has utilized FTIR microscopy to quantitatively map the spatial distribution of proteoglycan and collagen content in native,^8^, ^9^, ^12^, ^14^, ^18^, ^24^ osteoarthritic cartilage,^18^, ^25^, ^26^ enzymatically‐degraded,^24^ and tissue‐engineered cartilage.^14^, ^22^ IVD tissue has a similar biochemical constituents to articular cartilage, however the ratio of proteoglycans and collagen is varied across the NP and AF.^5^ Thus, methods must be developed that can accurately measure biochemical components in both the proteoglycan‐rich NP and collagen‐rich AF.

Here we present FTIR microscopy with peak integration as a method to quantitatively assess the local distribution of biochemical composition of the entire IVD. This method is useful because it can be performed on unstained histological sections and does not require a priori knowledge of which components are to be analyzed. This method can be used by investigators across IVD development, disease progression and regenerative therapies to quantify local changes to IVD biochemical content. This technique was developed using sheep lumbar IVDs, but can be applied to any spine level or animal species. The method was validated by comparing healthy lumbar IVDs to those that were injured via a discectomy to induce degenerative changes. The objectives of this study were: (a) to develop FTIR microscopy methods to analyze IVD tissue, (b) compare collagen and proteoglycan maps in native and herniated IVDs, and (c) compare patterns of collagen and proteoglycans with known grades of degeneration.

## 2 MATERIALS AND METHODS

### 2.1 Tissue source and preparation

Sheep lumbar IVDs were chosen as models of the human lumbar spine due to the anatomical, mechanical, and biochemical similarities between the two species.^27^, ^28^ A total of 13 healthy and degenerated sheep IVDs (Ironwood Hill Farm, Newark Valley, New York) were obtained from a previously performed in vivo study in which degeneration was induced in some levels via discectomy.^29^, ^30^ As described previously, the lumbar spines of six Finn sheep were exposed through a lateral pre‐psoas approach.^29^, ^31^ One to two lumbar IVDs per animal were subjected to a 3 × 10 mm annulotomy via scalpel followed by removal of ~200 mg of NP tissue using pituitary rongeurs. This has been shown in previous studies to induce degenerative changes including loss of hydration, disc height, mechanics and biochemistry over a six‐week period in vivo.^29^ One IVD per animal served as an intact control and did not receive the discectomy. After 6 weeks in vivo, all spines were harvested, subjected to magnetic resonance imaging (MRI) in the sagittal plane using a 3 Tesla scanner (Siemens, Erlangen, Germany), and the IVDs were prepared for histological sectioning. Three physicians blinded to the treatment groups used the Pfirrmann grading system to analyze the state of IVD degeneration on a scale from 1 to 5 with respect to the signal intensity and homogeneity of the NP as well as the distinction of the border between NP and AF on sagittal and axial T2 MRI sequences. The reported Pfirrmann grade for each IVD was taken as the median of the three grades. The sheep study was approved by the Cornell University and the Barton West End Farms Institutional Animal Care and Use Committee and was performed according to the guidelines recommended by these committees.

### 2.2 Histological evaluation

All IVDs were fixed with 10% neutralized formalin buffer, decalcified in 5% nitric acid for 1 to 2 months, and transferred to 75% ethanol prior to paraffin embedding. Samples were sectioned at 4 μm thickness through a mid‐coronal plane onto glass slides. After sectioning onto glass slides, samples were stained with alcian blue and imaged with brightfield microscopy with an Aperio slide scanner (Leica Biosystems, Buffalo Grove, Illinois).

### 2.3 FTIR microscopy and analysis

**FIGURE 1:**
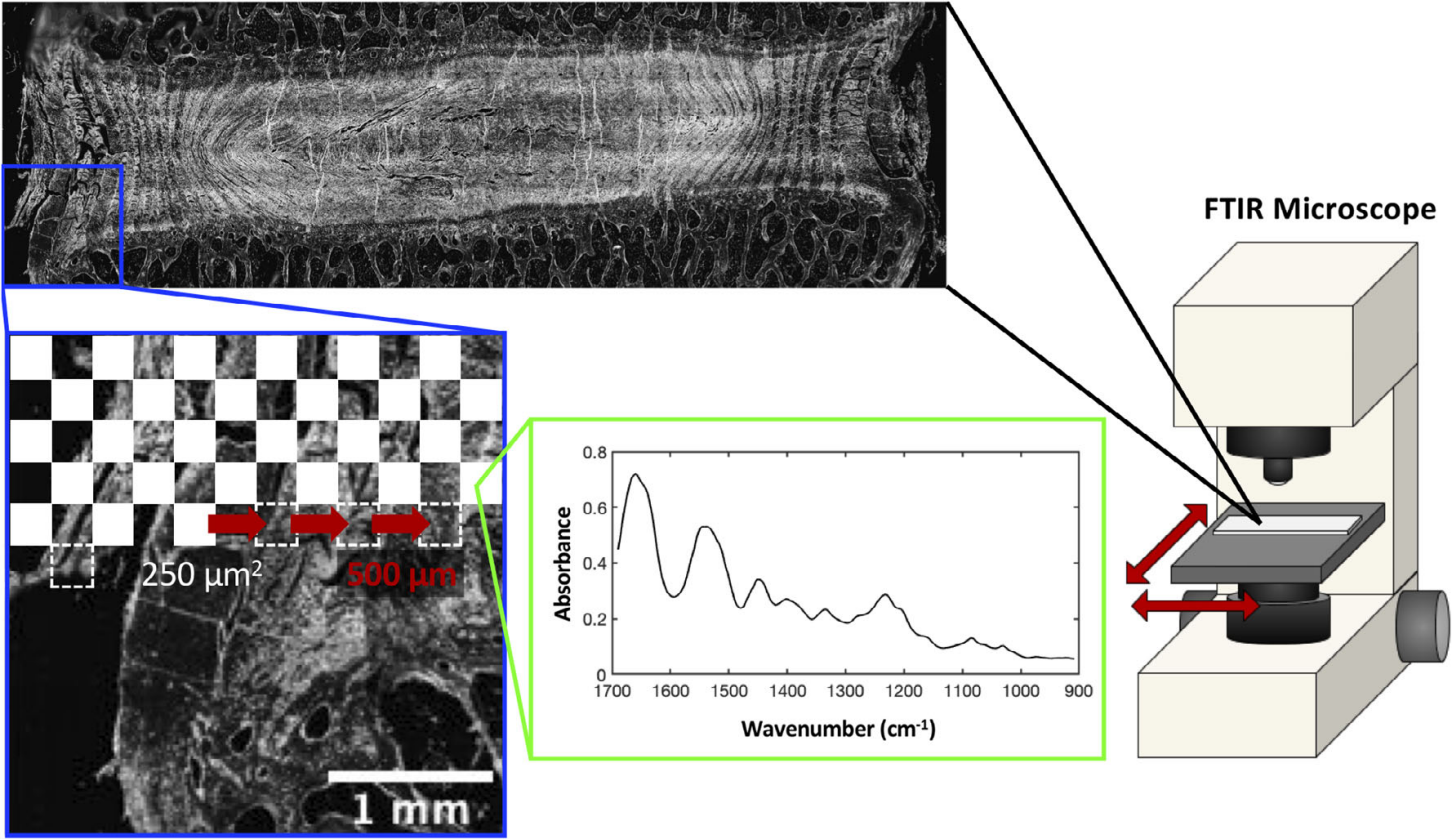
A schematic representation of the Fourier transform infrared (FTIR) microscopy imaging setup and acquisition methods. intervertebral disc (IVD samples mounted on barium fluoride slides were imaged with a Hyperion 3000 FTIR microscope under a 15× IR‐compatible objective. A brightfield overview image was obtained in order to visualize the region of interest of the scan. Then, hundreds of FTIR spectra were obtained across a rectangle containing the annulus fibrosus, nucleus pulposus, and cartilage endplates. The individual scans were obtained over a 250 μm^2^ area and spaced 500 μm apart

**FIGURE 2:**
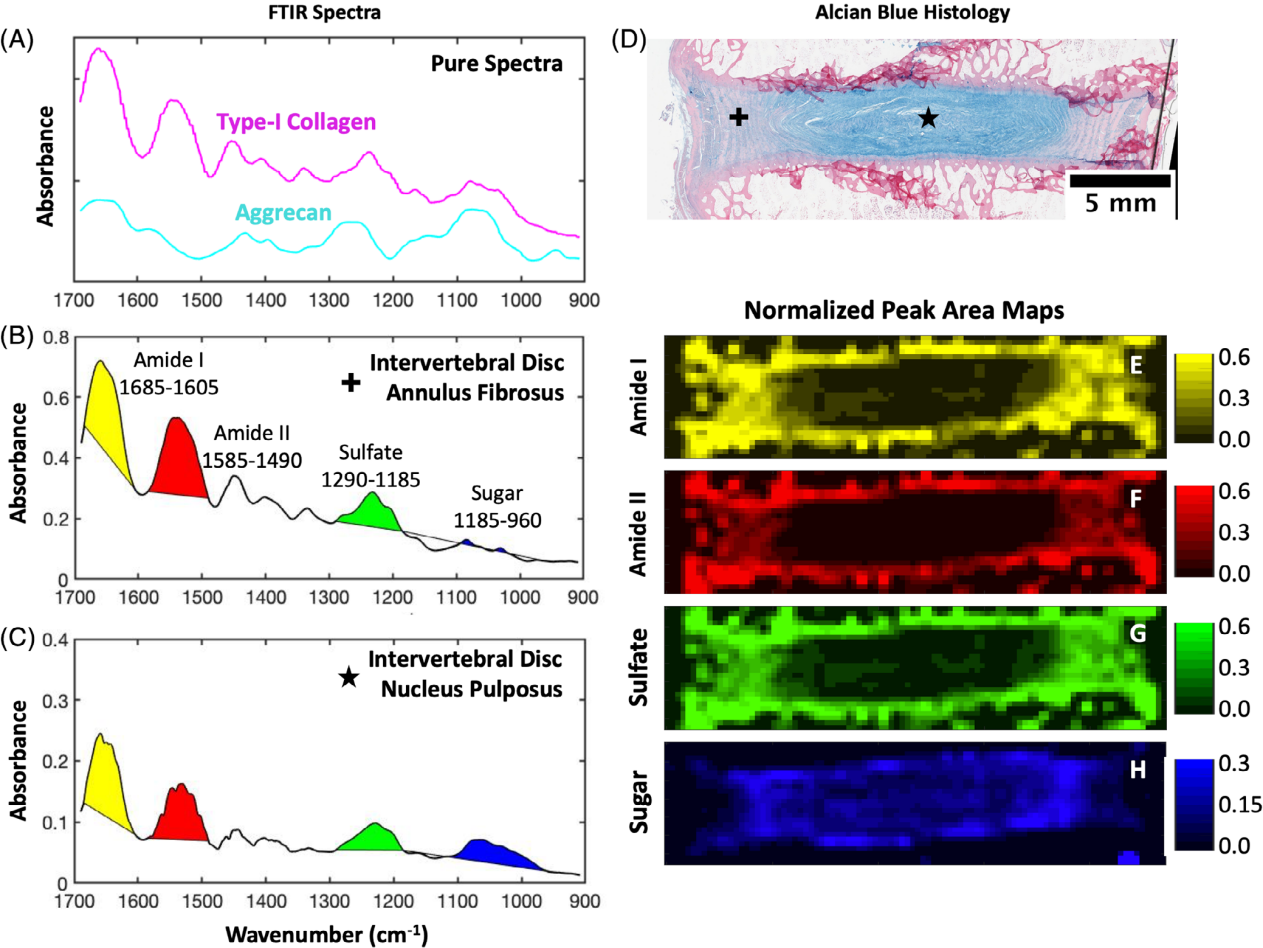
Representative Fourier transform infrared (FTIR) spectra obtained from A, pure type‐I collagen and aggrecan (from a previous study^12^), B, annulus fibrosus tissue and C, nucleus pulposus tissue show unique spectral characteristics. Peak areas were obtained from the FTIR spectra by integrating between wavenumbers associated with the with the amide I, amide II, sulfate and sugar regions. D, Representative alcian blue histology of an intact intervertebral disc (IVD) showing the location of where FTIR spectra in panels B and C were taken from. Proteoglycans are stained blue and collagen pink. E‐H, Representative peak area maps of the amide I, amide II, sulfate, and sugar peaks from the intact IVD in D

Following a modified protocol that was previously established for articular cartilage,^9^ 4 μm thick histological sections prepared as described above were placed on 3 mm thick IR‐transparent barium fluoride slides that were 32 mm in diameter (Spectral Systems, Hopewell Junction, New York). Sections were deparaffinized in xylene and rehydrated in successive 100%, 95%, and 70% ethanol baths. Samples were imaged with a Hyperion 3000 FTIR microscope (Bruker, Billerica, Massachusetts) under a compressed air purge to evacuate excess water vapor. Prior to FTIR imaging, a brightfield overview image was obtained in order to determine the rectangular region of interest (ROI) that contained the IVD, endplate and a small area of each adjacent vertebrae (Figure 1). In transmission mode, FTIR sample absorbance spectra were obtained with a resolution of 4 cm^−1^ using an IR‐compatible 15X objective. Spectra at each point were averaged over 16 background‐corrected scans between 600 and 4000 cm^−1^. The microscope rastered across the rectangular ROI (~1.5 cm^2^) by collecting FTIR spectra over individual tiles that were 250 μm^2^ in area and spaced 500 μm apart (Figure 1). A single element Mercury‐Cadmium‐Telluride (MCT) detector was used in conjunction with a scanning microscope stage for image acquisition. The step size and the aperture size were both set to 250 μm, resulting in a pixel size of 250 μm × 250 μm. FTIR spectra for pure type‐I collagen and aggrecan were obtained from a previously reported study^12^ in order to compare IVD spectra with pure ECM components (Figure 2A‐C).

The amide I, amide II, sulfate, and sugar peaks from each FTIR spectra were integrated between 1605‐1685 cm^−1^, 1490‐1585 cm^−1^, 1185‐1290 cm^−1^, and 960‐1185 cm^−1^, respectively. These values were chosen based on previously reported values for articular cartilage,^8^, ^9^, ^14^, ^15^, ^16^, ^17^ but were optimized to match the peaks found in IVD tissue (Figure 2B,C). Peak integration was performed by plotting a line between the intersection of the FTIR spectrum and the given wavenumber range for each peak, and then integrating the area between the line and the spectrum as described previously.^8^, ^14^, ^16^, ^17^, ^22^ Only positive area was included in the peak area measurements. Spectra were not baseline corrected prior to peak integration, but the method of peak integration effectively baselines each peak. The four peak areas at each raster tile were then divided by the mean of the respective 10 highest peak areas from the same IVD to account for differences between animals and lumbar levels. Peak area maps were generated from the normalized peak areas for the amide I, amide II, sulfate, and sugar peaks and compared to alcian blue histology (Figure 2D‐H). Peak area values were interpolated over unsampled areas using bicubic interpolation. To quantify spatial differences in peak areas between intact and discectomized IVDs, peak area values were collected from line scans across the width of each IVD. IVD width was normalized to 1, and average peak area values were averaged together for intact and discectomized IVDs. To validate the FTIR findings against the known bulk biochemical composition of intact and degenerated IVDs, average peak areas were calculated in the NP and AF regions from both intact and discectomized IVDs. Regions of NP and AF were manually selected with elliptical ROIs, and peak area values within these ROIs were averaged.

### 2.4 Statistical methods

A two‐way analysis of variance (ANOVA) was used to determine statistical differences in the ROI average peak area plots, which accounted for tissue region (NP or AF) and tissue type (intact or discectomy). Multiple comparisons were made for the sugar and amide II average peak areas between tissue type and region using two‐tailed Tukey's honest significant difference post hoc tests. Spearman's rank correlation coefficients were used to assess statistical significance for correlation plots between peak area and Pfirrmann grade. *P* values less than .05 were considered statistically significant.

## 3 RESULTS

IVD tissue had unique, location‐dependent FTIR spectral characteristics across the AF and NP, and showed absorbances typical of both collagen and proteoglycans. Pure type‐I collagen showed notable peaks in the amide I, amide II, and sulfate regions (Figure 2A). The amide I region is the most intense absorbance band in proteins, and is mostly present due to stretching vibrations of the C=O and C—N groups.^8^, ^23^ The amide II region has prominent absorbance due to N—H bending as well as C—N and C—C stretching vibrations. The amide III and sulfate regions heavily overlap from 1290 to 1185 cm^−1^, which explains why collagen showed high absorbance in this region even though the pure protein does not contain sulfate groups. Pure aggrecan also had notable peaks in the amide I and sulfate regions, but more interestingly lacked absorbance in the amide II region and had a large peak in the sugar region (Figure 2A). Based on these data, the amide II peak was chosen to be representative of collagen, while the sugar peak was chosen to be representative of proteoglycans. When FTIR spectra were collected from IVD tissue, AF and NP tissue had spectral similarities to collagen and aggrecan, respectively. FTIR spectra from the outer AF region of the IVD showed prominent peaks in the amide I, amide II, and sulfate regions, with little absorbance in the sugar band (Figure 2B). NP tissue had lower overall IR absorbance than AF tissue, and shows distinct peaks in the amide I, amide II, sulfate, and sugar regions (Figure 2C).

Maps generated from peak area measurements showed location‐dependent changes across the IVD in the amide I, amide II, sulfate, and sugar regions, and showed unique features compared to alcian blue histology (Figure 2D‐H). Amide I, amide II, and sulfate peak area maps all showed similar features, with high absorbance in the outer AF and endplate, and lower absorbance in the inner AF and NP (Figure 2E‐G). These three IR bands showed decreasing absorbance from the outer AF to inner AF, which matches the decreasing proteoglycan content visible in the alcian blue histology and what is known about IVD composition.^32^, ^33^, ^34^, ^35^ The amide II peak area map showed the least amount of absorbance in the NP and inner AF regions, since proteoglycans such as aggrecan do not have high IR absorbance in that region. The sugar peak area map was the most unique of the four measured in this study, showing high absorbance in the NP and inner AF regions and little absorbance elsewhere (Figure 2H).

There are two noteworthy differences between proteoglycans visualized through alcian blue histology and FTIR microscopy. First, the distribution of proteoglycan‐rich tissue appeared fairly homogenous across the NP and inner AF through FTIR microscopy. In alcian blue histology the most intense staining of proteoglycans was found in the NP, which then decreases across the inner AF to the outer AF. Second, FTIR microscopy revealed a larger region containing proteoglycans than what is visualized through histology.

**FIGURE 3:**
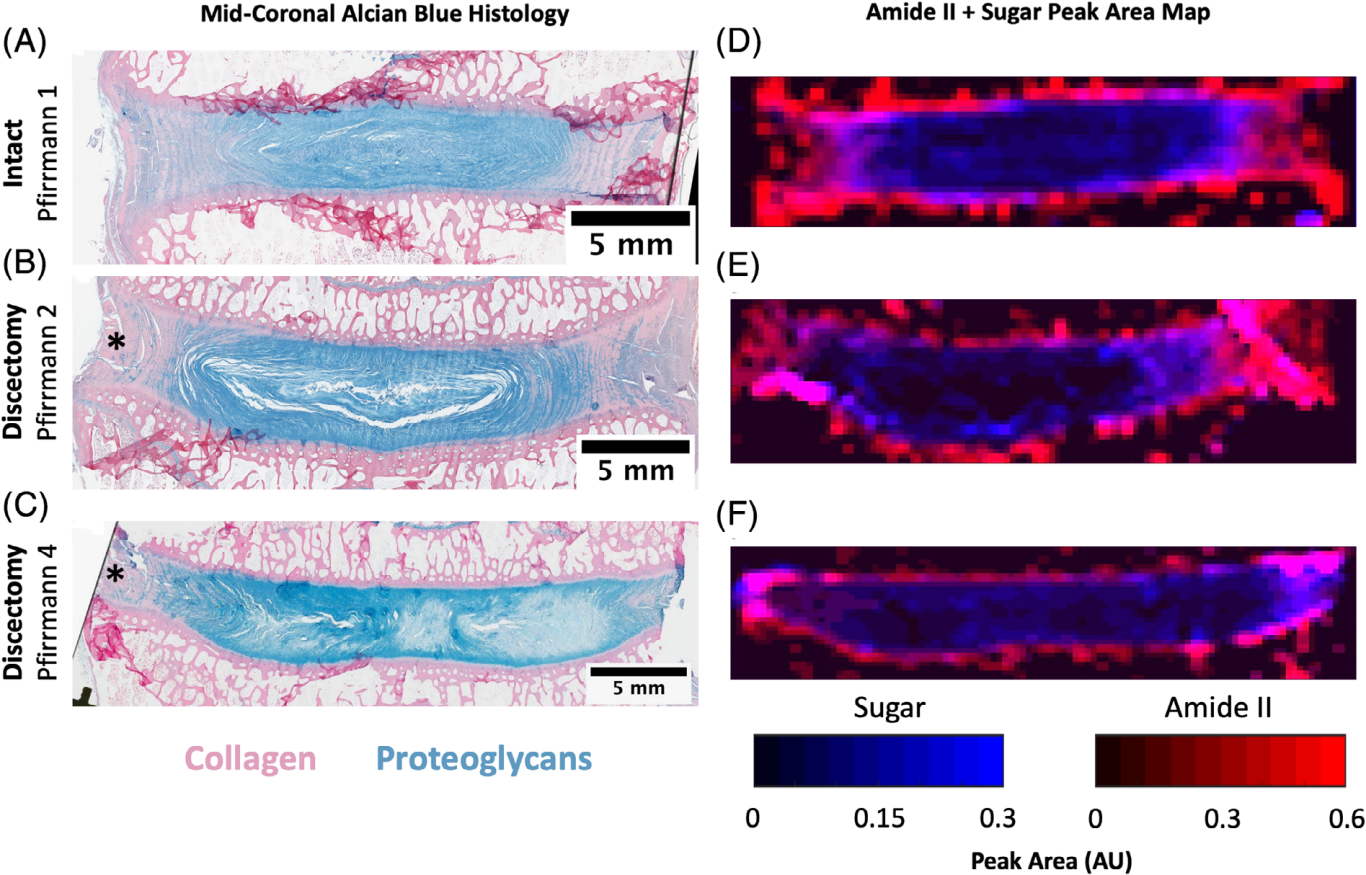
Representative alcian blue histology of intact and discectomized intervertebral discs (IVDs) compared to amide II/sugar peak area maps from the same IVDs. A, An intact IVD that showed no signs of degeneration on MRI (Pfirrmann grade 1), has a homogenous nucleus pulposus (NP) that stains blue for proteoglycans, and an annulus fibrosus (AF) that stains mostly pink for collagen. B, A representative discectomized IVD with mild degeneration (Pfirrmann grade 2) showing some fibrosity and disruption in the nucleus pulposus (NP) and C, a discectomized IVD with greater degeneration (Pfirrmann grade 4) with a heterogeneous NP and increased proteoglycan staining in the AF. D‐F, Peak area maps of the combined amide II and sugar peak areas show the quantitative distribution of collagen (red) and proteoglycans (blue) from Fourier transform infrared microscopy of each IVD from panels A‐C. The intact IVD map shows homogenous proteoglycan distribution, while the discectomized IVD maps are more heterogeneous and show a reduction in proteoglycans on the ipsilateral side to the discectomy injury. Asterisks indicate the ipsilateral side to the discectomy injury

Alcian blue histological sections of intact and discectomized IVDs were compared to data generated from FTIR microscopy to evaluate how each method can be used to study local changes in biochemical content. As mentioned above, in intact IVDs with no signs of degeneration, alcian blue staining showed proteoglycans primarily localized to the NP (Figure 3A). While there was evidence of proteoglycans in the AF, most of the AF stained pink for collagen. In discectomized IVDs with low Pfirrmann grades of degeneration, the blue stain for proteoglycans was similarly localized to the NP with slightly more proteoglycans in the AF (Figure 3B). The NP appeared less homogenous than the intact IVDs, appearing disrupted with some fibrous tissue. IVDs with high Pfirrmann grades had a highly heterogeneous NP with regions of dark and light blue staining for proteoglycans (Figure 3C). Compared to intact controls, the NP was much more fibrous and the AF shows more blue staining. FTIR peak area maps distinguished local changes in collagen and proteoglycans that the alcian blue histology does not show (Figure 3D‐F). As described above, FTIR microscopy showed that intact IVDs have a homogenous proteoglycan‐rich region that is larger than observed with histology (Figure 3D). In discectomized IVDs with low and high Pfirrmann grades, FTIR microscopy showed heterogeneity in the proteoglycan‐rich region of the IVD and less collagen content in the AF compared to intact controls. This technique also revealed a reduction in proteoglycans in the NP/AF region ipsilateral to the discectomy injury. This observation is notable because the alcian blue stain does not show reduced proteoglycan content on the side that received the discectomy.

**FIGURE 4:**
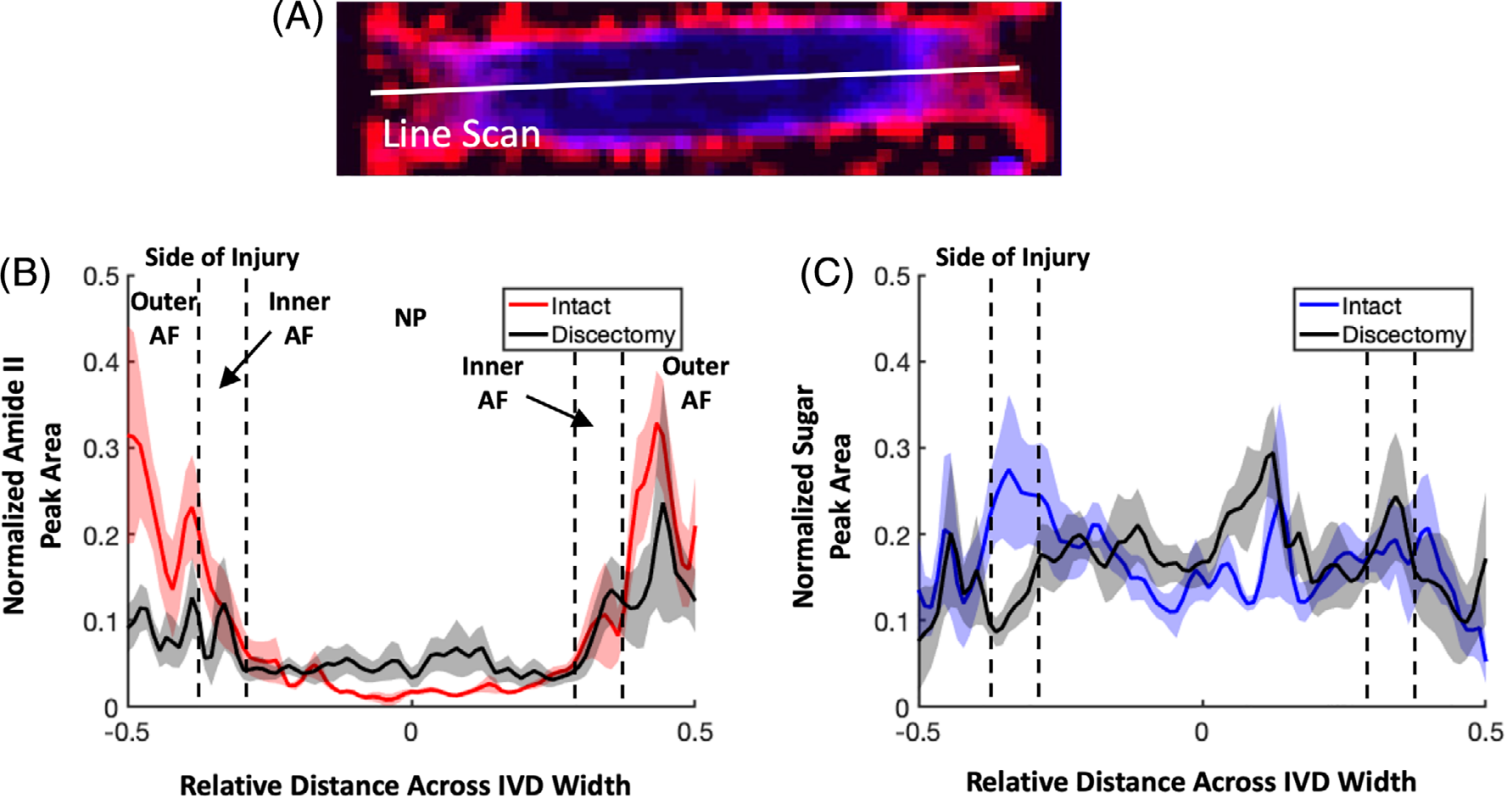
A, Peak area values were collected from line scans across the width of each intervertebral disc (IVD). IVD width was normalized to 1, and average peak area values were averaged together for intact and discectomized IVDs. B, Average normalized amide II and C, sugar peak area plots showing the average ± SE of the mean across the width of the IVDs. Dotted lines show approximate borders of the outer annulus fibrosus (AF), inner AF and nucleus pulposus. The sample size was 6 to 7 samples per group

To quantify local differences in the collagen and proteoglycan composition of intact and discectomized IVDs, peak area values were collected from line scans across the width of each IVD (Figure 4A). The width of each IVD was normalized to 1, and then peak areas from each IVD within the intact and discectomy groups were averaged. The average line scan of amide II peak area in intact IVDs had a symmetrical shape, with high peak area in the outer AF that diminished by more than 90% in the NP (Figure 4B). Discectomized IVDs had lower amide II peak area on the side of the discectomy, increased peak area in the NP, and similar peak area to intact IVDs on the contralateral side to the discectomy. The average line scan of sugar peak area had a less distinct shape, but peak area appeared lower on the periphery of the IVD and higher in the NP region (Figure 4C). Differences between intact and discectomy IVDs were confined to the inner AF on the side of the defect and a small region near the center of the NP.

**FIGURE 5:**
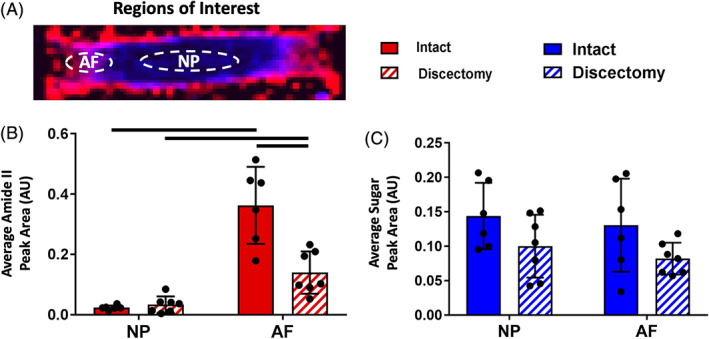
A, Average peak area values were quantified from regions of interest in the nucleus pulposus and annulus fibrosus (AF) for intact and discectomized intervertebral discs (IVDs). The AF region of interest for discectomized IVDs was taken ipsilateral to the discectomy injury. Average peak area plots of B, amide II and C, sugar peaks. The sample size was 6 to 7 samples per group, error bars are ±SD, and bars above groups denotes statistically significant differences at *P* ≤ .05. A two‐way ANOVA with two‐tailed Tukey's honest significant difference post hoc tests was used to assess statistical differences

In order to validate the FTIR method against literature reports of IVD biochemical composition in intact and degenerated states, aggregate peak area values were gathered in the AF and NP from intact and discectomized IVDs. Once regions were selected from defined ROIs in the AF and NP (Figure 5A), the amide II and sugar peak areas were averaged and compared. Representative of collagen content, the average amide II peak areas were higher in the AF region compared with the NP region of intact IVDs (Figure 5B). In both the intact and discectomized IVDs, the AF region had significantly higher average amide II peak area than NP tissue (intact *P* = .0012 and discectomy *P* = .006). Comparing intact and discectomized IVDs, the NP regions had similar average amide II peak area, but the AF were significantly different with the intact and discectomy having a mean of 0.36 ± 0.13 and 0.14 ± 0.07, respectively (*P* = .006). The average sugar peak area, representative of proteoglycan content, had greater variation than the amide II peak area in both the NP and AF (Figure 5C). No significant differences found between the intact and discectomized IVDs, or between the AF and NP regions.

**FIGURE 6:**
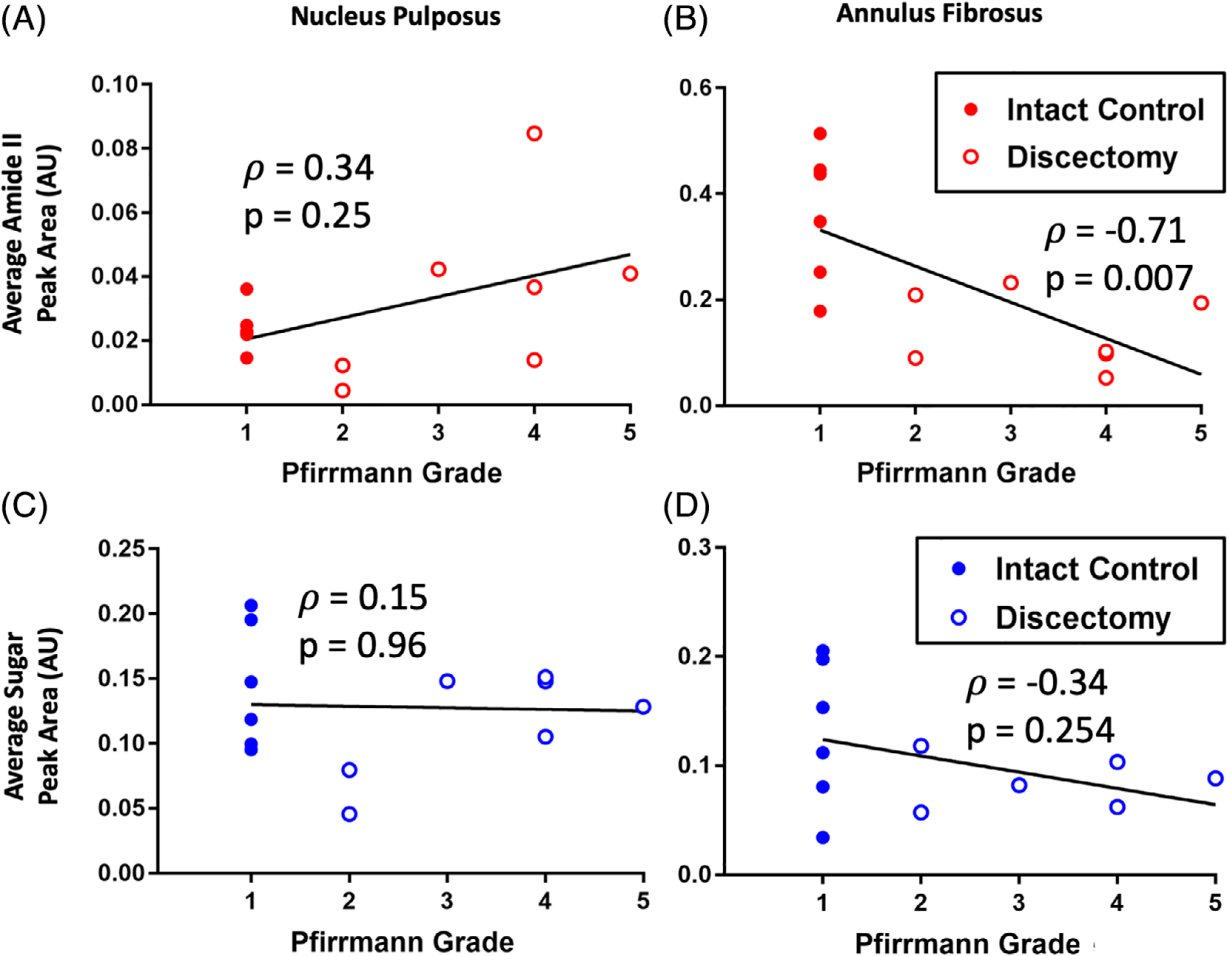
A‐D, Correlations comparing average peak areas from Fourier transform infrared microscopy to intervertebral disc (IVD) degeneration via Pfirrmann grading in the nucleus pulposus (NP) and annulus fibrosus (AF). The strongest correlation was found between the average amide II peak area in the AF and Pfirrmann grade, with a Spearman *ρ* value of −0.71 (*P* = .007). The weakest correlation was found between average sugar peak area in the NP and Pfirrmann grade, with a *ρ* value of 0.15 (*P* = .96). The total sample size for each plot is 13 IVDs, and Spearman's rank correlation coefficients were used to assess statistically significant differences at *P* ≤ .05

The FTIR biochemical analyses were further validated by investigating correlations between the average peak areas and Pfirrmann grade. The Pfirrmann grade is a semi‐quantitative grading scale used clinically and in pre‐clinical large animal models to describe IVD degeneration on a scale from 1 to 5 based on sagittal MRI (1‐least degeneration, 5‐most degeneration). In this study, all the intact IVDs had a Pfirrmann grade of 1 since they did not suffer any injury, while all of the discectomized IVDs had Pfirrmann grades ranging from 2 to 5 (Figure 6). The average amide II and sugar peaks areas from the NP and AF showed strong correlations with Pfirrmann grade, except for the average sugar peak area from the NP (Figure 6A‐D). The strongest correlation to Pfirrmann grade was the average amide II peak area in the AF, with a Spearman *ρ* value of −0.71 (*P* = .007). The weakest correlation to Pfirrmann grade was average sugar peak area in the NP, with a *ρ* value of 0.15 (*P* = .96).

## 4 DISCUSSION

The objectives of this study were: (a) to develop FTIR microscopy methods to analyze IVD tissue, (b) compare collagen and proteoglycan maps in native and herniated IVDs, and (c) compare patterns of collagen and proteoglycans with known grades of degeneration. The data from this study showed that FTIR microscopy enabled quantitative measurements of local IVD composition, and quantitative comparison of local composition between intact and discectomized IVDs. AF and NP tissues had unique FTIR spectra with respect to the amplitude and wavelength of IR light absorbance peaks. Peak area maps of the amide II and sugar absorbance revealed a unique biochemical distribution compared with traditional histology stains. Also, since IR light absorbance is linear and Beer's law states that absorbance directly correlates with molecular concentration, FTIR microscopy is a highly quantitative method in comparison to traditional histology. This technique was validated by comparing aggregated IR absorbance from the AF and NP regions from intact and discectomized IVDs against traditional histology and what is known about the IVD in healthy and degenerated states. Lastly, the aggregated FTIR absorbances for collagen and proteoglycans correlated well with IVD degeneration scored through Pfirrmann grades.

The data from this study are consistent with previous investigations of human IVD biochemical composition that use wet chemistry assays to quantify biochemical content. Literature investigations of the collagen and proteoglycan content of the IVD in healthy and degenerated states have used hydroxyproline assays to measure collagen content and dimethylmethylene blue or uronic acid assays to measure proteoglycan content.^32^, ^33^, ^34^, ^36^ Due to the nature of wet chemistry assays, spatial data is discrete but some investigations gathered tissue from multiple locations across the IVD. These studies showed that healthy IVDs have the highest proteoglycan content in the NP and inner AF, which decreases approximately two‐ to threefold towards the outer AF.^32^, ^33^, ^35^ Collagen content is the highest in the periphery of the IVD, and decreases two‐ to threefold towards the center of the NP.^32^, ^33^ These results are consistent with the data produced by FTIR microscopy in Figures 4 and 5 of the present study, but the present FTIR data show a larger change in collagen content and lesser change in proteoglycan content between the AF and NP. Previous studies also showed that in a degenerated state, collagen content is increased 0% to 100% in the NP and decreased approximately 10% to 35% in the AF compared to healthy controls.^32^, ^33^ Lastly, proteoglycan content in all IVD regions is decreased about 10% to 50% in a degenerated state.^32^, ^33^, ^36^ In the present study, FTIR microscopy showed that collagen content is increased ~40% in the NP and decreased ~60% in the AF of discectomized IVDs, fairly consistent with the literature on human IVDs. The current study did not show differences in proteoglycan content between healthy and injured IVDs. The discrepancies between this study and previous reports of proteoglycan content could be due to the lower levels of degeneration seen in IVDs in the present study, and differences in sheep vs human IVD biochemistry.

Sheep lumbar spines were used in this study due to their mechanical and biochemical similarities to humans.^28^, ^37^, ^38^ Reports have shown that sheep have a similar range of motion and intradiscal pressure to human spines, even though sheep are quadrupeds and have a horizontal spine. Previous studies have also reported that sheep and humans have similar spatial distribution of biochemical contents across the IVD, and accumulate degenerative changes in a similar manner with age.^27^, ^37^, ^39^ Humans, sheep, cows and other large animal models lose notochordal cells as they age, which leads to loss of proteoglycan content in the NP with age. Other animal models, such as pig and rat spines, maintain their notochord through adulthood and maintain a proteoglycan‐rich, fluid NP into adulthood.

This study is not the first to report a method for imaging the biochemical content of the IVD with FTIR microscopy, but presents an optimized method that yields high quality data across the entire IVD. There are a limited number of studies reporting FTIR microscopy on IVD tissue. Mader et al imaged intact and enzymatically degenerated IVDs, and used a multivariate analysis of the second derivative of the FTIR spectra to parse out factors that relate to type‐I collagen, type II collagen, elastin, and chondroitin sulfate content.^11^ The reliability of distinguishing between unique components based on second derivatives is not definitive due to the overlap in IR absorbance, but strong correlations were found between markers of degeneration and FTIR proteoglycan data. Hadjab et al used FTIR to analyze elastin, collagen, and proteoglycan content in intact and degenerated IVDs^40^. It is unclear what method was used to obtain component absorbance or where scans were taken, and the only data presented are bar plots of quantified absorbance. Southern et al used FTIR microscopy to assess biochemical changes to the IVD after intradiscal electrothermal annuloplasty (IDET).^10^ FTIR spectra were analyzed for shifts in the amide I contour and a ratio of the amide II to sulfate peak areas, but the study only reported spectra in small regions of the AF near IDET treatment. There are a larger number of studies employing FTIR microscopy to analyze biochemical content of articular cartilage, but these studies are difficult to compare to due to the difference is biochemistry from IVD tissue.^8^, ^9^, ^13^, ^16^, ^24^, ^25^, ^26^, ^41^ While these studies present methods that work well for articular cartilage, the analytical techniques are optimized for collagen‐rich matrices and not the aggrecan‐rich NP unique to the IVD. Compared to previous work, the present study describes an optimized imaging setup for whole IVD tissue with an analytical method that yields quantitative biochemical data across the entire IVD. This study also reports novel biochemical composition maps of the whole IVD in healthy and degenerated states.

Raman spectroscopy has also been used as a quantitative method to measure the biochemical content of the IVD and cartilaginous tissue.^19^, ^20^, ^42^ Raman spectroscopy is a similar technique to FTIR spectroscopy, but detects light scattering from vibrating molecules rather than detecting IR absorbance.^43^ As such, Raman spectroscopy can image hydrated, unfixed tissue and directly measure local water content. Compared to FTIR methods, Raman spectroscopy is well‐suited for analyzing inorganic and crystalline components.^43^ Recent studies have measured significant differences in the water: protein ratio between the AF and NP, and also revealed lipid composition of the AF.^20^ Raman spectroscopy is complementary technique to FTIR microscopy, and may provide improved physiologic compositional data because it can be performed on hydrated, unfixed tissue.

FTIR microscopy has many benefits over traditional histology and IHC that stem from IR absorbance physics vs dye‐binding kinetics. Traditional histology is widely accepted and slightly easier to perform, however dye‐binding kinetics are non‐linear and thus true biochemical composition cannot be accurately visualized or quantified.^44^ This explains why the alcian blue images showed a smaller proteoglycans‐rich area compared to the FTIR sugar peak area maps in Figure 3. The NP is predominantly proteoglycans while the AF has a mixture of collagen and proteoglycans, thus the alcian blue dye will behave differently in the two tissues because the affinity of a pure proteoglycan matrix is different than proteoglycans within a collagen matrix. FTIR is able to parse out the direct concentration of each biomolecule because IR absorbance increases linearly with concentration. Another benefit stems from FTIR microscopy not requiring the investigator to know a priori what biochemical constituents they will be analyzing spectra for. FTIR microscopes simply provide IR absorbance of a particular tissue, and thus the spectra can be examined for any number of biomolecules. This contrasts with histology, IHC and spectrophotometric assays, where only one or a handful of components can be analyzed on a single slide or sample. Additionally, there are many errors introduced in traditional biochemical analysis methods such pipetting, the age of stock solutions, timing of washes and mass measurements that can skew results from reality. With FTIR microscopy, once an algorithm is written to parse out various biochemical components from FTIR spectra, it can be used infinite times with no user error.

One last major benefit of FTIR microscopy is its quantitative nature. As seen in Figure 4, quantitative analyses can be performed to evaluate both the concentration and spatial distribution of biochemical components within the IVD. The analyses in this study showed that discectomized IVDs had a multiple‐fold increase in collagen content in the NP compared to intact controls. This local change in NP biochemistry cannot be seen nor quantified through alcian blue histology. FTIR microscopy can identify small changes in biochemical concentration that may be undetectable with other methods. Lastly, the analysis presented in Figure 4 is one of many novel quantitative analyses that may be used with FTIR spectral maps.

While FTIR microscopy is a promising method for quantifying the local biochemical content of the IVD, there are limitations that must be addressed with regards to the spectral analysis and tissue processing. First, some bond vibrational modes have overlapping absorbance bands, as seen with amide III and sulfate IR absorbance. This is likely why the peak area analyzed in the 1185 to 1290 cm^−1^ range resulted in strong absorbance in the AF from collagen when the wavenumber range is also known for sulfate absorbance associated with proteoglycans. Peak area measurements are sensitive to the bounding wavenumber ranges, and the method presented here describes ranges optimized for IVD tissue. Second, the FTIR spectra can be analyzed using a variety of methods that yield similar yet varied results. In this study, we chose to analyze peak areas by integrating between known wavenumber ranges, but other prominent analytical methods utilize linear decomposition,^9^ first and second derivatives,^11^ peak height,^45^ Gaussian curve fitting,^46^ and peak area with varied integration techniques,^14^, ^15^, ^16^ and multivariate analyses.^11^, ^47^ Each of these methods have their own benefits and limitations. Future studies may wish to quantify the accuracy of these methods using controlled depletion of ECM molecules. Third, studies have shown that FTIR absorbance is non‐linear in some biological tissues over small length scales (~30 μm) due to Mie scattering from cells.^48^ This is not a major concern to this study, however, as FTIR absorbance was collected over larger areas (250 μm × 250 μm) and the peak integration method effectively baselines each measurement. Lastly, as with other histology‐based methods, artifacts can be introduced through processing and sectioning that alter the specimen such as loss of tissue, tissue folding and fracture. Histological artifacts can be controlled to a degree by excluding regions with known artifact from analysis.

## 5 CONCLUSION

FTIR microscopy with peak area integration is a highly quantitative method to analyze the local biochemical content of the IVD. This study demonstrated that IVDs have unique FTIR spectra in the AF and NP that can be used to construct maps to show the spatial distribution of collagen and proteoglycans. Compared to traditional histology stains, FTIR microscopy enables direct quantification and visualization of biomolecules within the IVD without a priori knowledge of the constituents. This technique is widely applicable in the field of IVD research, and may help investigators to better understand and quantify the local distribution of biomolecules in development, disease progression and repair.

## CONFLICT OF INTEREST

Roger Härtl is a consultant for AO Spine, Brainlab, Depuy‐Synthes and Lanx, and received research funding from Baxter. Lawrence J. Bonassar is a consultant for Fidia Pharmaceuticals and 3DBio Corp.

## AUTHOR CONTRIBUTIONS

Stephen R. Sloan: study design, data acquisition and analysis, interpretation of results, and manuscript preparation. Christoph Wipplinger: interpretation of results and manuscript preparation. Sertaç Kirnaz: interpretation of results and manuscript preparation. Robert Delgado: data acquisition, interpretation of results, and manuscript preparation. Steven Huang: data acquisition, interpretation of results, and manuscript preparation. Gennady Shvets: interpretation of results and manuscript preparation. Roger Härtl: interpretation of results and manuscript preparation. Lawrence J. Bonassar: study design, interpretation of results, and manuscript preparation.
